# BMP9 induces osteogenic differentiation through up-regulating LGR4 via the mTORC1/Stat3 pathway in mesenchymal stem cells

**DOI:** 10.1016/j.gendis.2023.101075

**Published:** 2023-09-07

**Authors:** Jie Zhang, Jinhai Jiang, Hang Liu, Shiyu Wang, Kaixin Ke, Siyuan Liu, Yue Jiang, Lu Liu, Xiang Gao, Baicheng He, Yuxi Su

**Affiliations:** a Department of Pharmacology, School of Pharmacy, Chongqing Medical University, Chongqing 400016, China; b Key Laboratory of Biochemistry and Molecular Pharmacology of Chongqing, Chongqing Medical University, Chongqing 400016, China; c Department of Orthopedics, Second Affiliated Hospital of Chongqing Medical University, Chongqing 400016, China; d Orthopedics Department, Children's Hospital of Chongqing Medical University, Chongqing 400014, China; e China International Science and Technology Cooperation Base of Child Development and Critical Disorders, Jiangxi Hospital Affiliated Children’s Hospital of Chongqing Medical University, Jiangxi 330000, China; f National Clinical Research Center for Child Health and Disorders, China

**Keywords:** BMP9, LGR4, mTORC1, Osteogenic differentiation, Stat3

## Abstract

Bone defects and non-union are prevalent in clinical orthopedy, and the outcomes of current treatments are often suboptimal. Bone tissue engineering offers a promising approach to treating these conditions effectively. Bone morphogenetic protein 9 (BMP9) can commit mesenchymal stem cells to osteogenic lineage, and a knowledge of the underlying mechanisms may help advance the field of bone tissue engineering. Leucine-rich repeats containing G protein-coupled receptor 4 (LGR4), a member of G protein-coupled receptors, is essential for modulating bone development. This study is aimed at investigating the impact of LGR4 on BMP9-induced osteogenesis in mesenchymal stem cells as well as the underlying mechanisms. Bone marrow stromal cells from *BMP9*-knockout mice exhibited diminished LGR4 expression, and exogenous LGR4 clearly restored the impaired osteogenic potency of the bone marrow stromal cells. Furthermore, LGR4 expression was increased by BMP9 in C3H10T1/2 cells. LGR4 augmented the benefits of BMP9-induced osteogenic markers and bone formation, whereas LGR4 inhibition restricted these effects. Meanwhile, the BMP9-induced lipogenic markers were increased by LGR4 inhibition. The protein levels of Raptor and p-Stat3 were elevated by BMP9. Raptor knockdown or p-Stat3 suppression attenuated the osteoblastic markers and LGR4 expression brought on by BMP9. LGR4 significantly reversed the blocking effect of Raptor knockdown or p-Stat3 suppression on the BMP9-induced osteoblastic markers. Raptor interacts with p-Stat3, and p-Stat3 activates the *LGR4* promoter activity. In conclusion, LGR4 boosts BMP9 osteoblastic potency in mesenchymal stem cells, and BMP9 may up-regulate LGR4 via the mTORC1/Stat3 signal activation.

## Introduction

Bone defects and delayed healing caused by trauma, tumors, infection, or congenital malformation are major challenges in orthopedic treatment.^1^ Bones have an inherent ability to regenerate, continue to reshape throughout life, and repair themselves to some extent. However, effective initiation of bone regeneration is necessary in cases where the natural self-repair mechanisms are insufficient.^2^ Tissue-engineered bone offers promising prospects for treating large bone defects and provides an effective approach to regenerative medicine.^3^ Mesenchymal stem cells (MSCs) are employed extensively in tissue engineering due to their self-renewal and multidirectional differentiation potential.^4^^,^^5^ Numerous routes drive the differentiation of MSCs into osteogenic lineages, and bone morphogenetic proteins (BMPs) have demonstrated tremendous potential as inducers of bone formation.^6^^,^^7^ Specifically, BMP9 has been shown to possess a significantly stronger osteogenic potential than other BMPs,^8^ and the concrete molecular mechanism has not been well-elucidated. Therefore, clarifying the details of osteogenic differentiation stimulated by BMP9 in MSCs may contribute to maximizing its osteogenic potential and accelerating bone tissue engineering development.

Wnt/β-catenin has been defined as an appealing target to regulate bone homeostasis and bone injury repair.^9^^,^^10^ β-Catenin serves a critical function in the transduction of Wnt/β-catenin signals.^11^ In MSCs, BMP9 amplifies the Wnt/β-catenin signal, and its osteogenic potency could also be markedly reduced by silencing β-catenin.^12^ Thus, Wnt/β-catenin is important for BMP9 to commit osteoblastic lineage from MSCs, although further exploration of the specific details is required. The Wnt/β-catenin signal is subject to regulation by various factors at multiple nodes. Leucine-rich repeats containing G protein-coupled receptor 4 (LGR4) functions as a mediator of signal transduction from the extracellular environment to the cytoplasm and plays important roles in several biological processes, including bone remodeling, immune responses, energy metabolism, and intestinal stem cell metabolism.^13^, ^14^, ^15^ *LGR4* nonsense mutation has been linked to low bone mineral density and osteoporotic fractures in bone metabolism.^16^ In mice, deficiency of *LGR4* results in impaired osteoblast differentiation, fetal bone formation, and postnatal bone remodeling, as well as inhibited bone fracture healing.^17^^,^^18^ Additionally, the aerobic glycolysis ability of osteoblasts was diminished when *LGR4* was deleted in bone marrow stromal cells (BMSCs).^19^ Wnt/β-catenin activity can be dramatically suppressed by *LGR4* knockout, and the LGR4 effect on osteogenesis is also partially regulated by Wnt/β-catenin.^20^^,^^21^ However, it is currently unclear whether LGR4 plays a role in the BMP9-induced activation of Wnt/β-catenin signaling.

The PI3K/Akt signal, which can be activated by glucose and a range of other growth factors or cytokines,^22^ is crucial in regulating the osteoblastic capability of BMP9 by directing the Wnt/β-catenin signal via GSK-3β.^23^^,^^24^ Nonetheless, the details about the crosstalk between PI3K/Akt and Wnt/β-catenin need further uncovering. PI3K/Akt regulates osteogenic differentiation through mTOR, however, the exact molecular mechanism remains unclear.^25^ There are two distinct protein complexes of mTOR, namely mTORC1 and mTORC2, which differ in their complex-specific subunits (Raptor and Rictor, respectively), although they share the common component mTOR.^26^^,^^27^ To date, the specific role of mTORC1 or mTORC2 in regulating BMP9-mediated osteogenesis has yet to be fully elucidated.

In this study, we determined the LGR4 possible role in regulating the potential of BMP9 for osteoblastic commitment, as well as investigated the possible crosstalk between mTOR and BMP9 to regulate LGR4 expression and Wnt/β-catenin activation. The findings may provide new insights into how BMP9 modulates the Wnt/β-catenin signal, which may advance bone tissue engineering development.

## Materials and methods

### Chemicals and reagents

Primary antibodies against BMP9 (sc-514211), LGR4 (sc-7974), Runt-related transcription factor 2 (RUNX2; sc-390715), osteopontin (OPN; sc-21742), phosphorylated signal sensor and activator of transcription 3 (p-Stat3, Tyr705; sc-8059), and β-actin (sc-47724) were purchased from Santa Cruz Biotechnology (Shanghai, China); antibodies against Rictor (27248-1-AP), Raptor (20984-1-AP), and PPARγ (16643-1-AP) were bought from Proteintech (Wuhan, China); antibody against Stat3 (A1192) was purchased from ABclonal Biotechnology (Wuhan, China). AG490 (HY-12000) was purchased from MedChemExpress (Shanghai, China).

*BMP9*-knockout (KO) mice (KOAIP210326YC2, No. 320818211100002054) were purchased from Cyagen Biosciences (Guangzhou, China), and 6-week-old male C57BL/6 mice (No. 0011229) required for calvarial bone defect assay were owned from the Animal Laboratory Center of Chongqing Medical University (Chongqing, China). The Ethics Committee of Chongqing Medical University gave its approval (No. 2022030) to all animal research. *BMP9*-KO heterozygote mice and wild-type (WT) mice from the same litter were used for experiments.

C3H10T1/2 (CCL-226), C2C12 (CRL-1772), MC3T3-E1 (CRL-2593), MEFs (SCRC-1008), and HEK293 (CRL-1573) cells were purchased from the American Type Culture Collection (Manassas, VA, USA). Primary BMSCs were isolated from the femurs and tibias of 4-week-old *BMP9*-KO mice and WT mice, respectively. Complete Dulbecco's Modified Eagle's Medium (DMEM) containing 10% fetal bovine serum, 100 U/mL penicillin, and 100 g/mL streptomycin was used to cultivate the cells at 37 °C in 5% CO_2_. The cell seeding density was 5 × 10^4^ cells/mL for alkaline phosphatase (ALP) staining (P0321S, Beyotime, Shanghai, China) and alizarin red S staining (MUBMD-90021, Cyagen, Beijing, China), or 5 × 10^5^ cells/mL for mRNA and protein extraction.

### Recombinant adenovirus construction

AdEasy system was used to construct the recombinant adenoviruses (Ad) for this research.^28^^,^^29^ Briefly, the mouse *BMP9* and *LGR4* coding sequences were obtained using PCR. The products and siRNA oligos for LGR4, Raptor, and Rictor were cloned into the shuttle vectors. The vectors were linearized and recombined homologously in BJ/5183 cells. Then, the viruses were packaged in HEK293 cells, and designated as AdBMP9, AdLGR4, AdsiLGR4, AdsiRaptor, and AdsiRictor. Green fluorescent protein (GFP) or red fluorescent protein (RFP) was used to tag viruses for tracking, and adenovirus expressing GFP (AdGFP) or RFP (AdRFP) alone was used as a control.

### Quantitative reverse transcription-polymerase chain reaction (RT-qPCR) assay

Following the manufacturer recommendations, TRIzol (15596018, Invitrogen, Carlsbad, California, USA) was used to extract total RNA from tissues and cells, and an RT kit (R037A, Takara, Dalian, China) cDNA was then used to produce cDNA. The qPCR assay was conducted in the Bio-Rad CFX Connect system (95 °C for 30 s; 95 °C for 5 s, 60 °C for 30 s, 40 cycles) with the support of the SYBR Green qPCR Mixture (B21202, Bimake, Shanghai, China). β-Actin was used as an internal reference gene. Relative mRNA expression was calculated by the ΔCt (Ct: cycle threshold) method as follows: relative expression = 2^–ΔΔCt^; ΔCt = Ct (target gene) − Ct (β-actin). Primers used for this study are shown in Table 1.

**Table 1:** Primers used for PCR assay.

Gene	Primer	Sequence (5′–3′)
*RUNX2*	Forward	GCCGGGAATGATGAGAACTA
	Reverse	GGACCGTCCACTGTCACTTT
*OPN*	Forward	GCAAACATCAGATCGTGCCC
	Reverse	CACCGCACTGTACACAGGAT
*LGR4*	Forward	TTGTTCATCACTGCCTGCCT
	Reverse	GCGCTCTCTGAGGAGAAGAC
*Raptor*	Forward	GACGGTTTTTGGACTTGGGC
	Reverse	CGTTGTCCTTCACGAGGTCA
*Rictor*	Forward	ATGACCGACCTGGACCCATA
	Reverse	AGTGTTCTGATTCGCCGGTT
*PPARγ*	Forward	TTTTCAAGGGTGCCAGTTTC
	Reverse	AATCCTTGGCCCTCTGAGAT
*β-acti*n	Forward	CCACCATGTACCCAGGCATT
	Reverse	CGGACTCATCGTACTCCTGC
*LGR4 (ChIP)* primer 1	Forward	GGAGGGGTCACGCTGTTTTA
	Reverse	TTGGTCAAGAGGAATGGGGC
*LGR4 (ChIP)* primer 2	Forward	ACACGTCTGAAAGCTGGAGG
	Reverse	CTGACTTGGCTGGATCTGCA
*LGR4 (ChIP)* primer 3	Forward	GGAGGGGTCACGCTGTTTTA
	Reverse	GTGACAAGGTTGTGCTGCTGCTG

### Western blotting and immunoprecipitation (IP) assay

Cells and tissues were subjected to treatment with radioimmunoprecipitation assay (RIPA) lysis solution (R0020, Solarbio, Beijing China) on ice for 30 min, and the protein samples were then collected after centrifugation for 15 min (12,000 *g*, 4 °C). The supernatant containing the proteins was collected and subsequently boiled for 10 min. The proteins were separated on polyacrylamide gels containing 10% sodium dodecyl sulfate before being transferred to a polyvinylidene difluoride membrane and then subjected to standard Western blot analysis. Finally, the proteins were visualized using a chemiluminescent kit (160072, Saimike Biotech, Chongqing, China). Data were collected and quantified using the Bio-Rad ChemiDoc XRS+ imaging system (Bio-Rad, Hercules, CA, USA).

For IP assay, RIPA lysis buffer containing protease and phosphatase inhibitors (B14002 and B15002, Bimake, Shanghai, China) was used to lyse cells on ice. Protein G magnetic beads (S1430, NEB China, Beijing, China) were pre-washed with 30 μL of lysis buffer. Cell lysates were incubated with Raptor primary antibody at 4 °C overnight. IgG was used as a negative control. The lysates were combined with 15 μL of pre-washed magnetic beads, which were then incubated at 4 °C for 1 h.

The complexes were gathered and rinsed with lysis buffer, and then 30 μL of lysis buffer was added to elute the proteins. Finally, a Western blot assay was performed using antibodies against p-Stat3 (Tyr705) and LGR4.

### Alkaline phosphatase (ALP) activity assay

C3H10T1/2 cells were plated at a density of 5 × 10^4^ cells/mL in 24-well plates. On days 5 and 7 after treatment, the ALP activity was measured according to instructions provided in the kit (C3206, Beyotime, Shanghai, China). Finally, the plates were scanned and quantified with the ImageJ software.

### Oil red O staining assay

C3H10T1/2 cells (5 × 10^4^ cells/mL) were seeded in 24-well plates, and the lipid formation was measured on day 7 after treatment. As directed by the kit (C0157M, Beyotime, Shanghai, China), the cells were fixed with oil red O fixing solution for 20 min before being rinsed twice with distilled water. After that, oil red O dye was applied to the cells for 20 min at room temperature. The plates were scanned and photographed after being thoroughly cleaned with distilled water twice.

### Mineralization assay

C3H10T1/2 cells (5 × 10^4^ cells/mL) were cultivated in complete DMEM with ascorbic acid (50 μg/mL), dexamethasone (10 nM), and β-glycerophosphate (10 mM). The mineralized nodules were visualized on day 21 with 0.4% alizarin red S solution (A5533-25G, Sigma–Aldrich, Beijing, China), and quantified using ImageJ software.

### Chromatin IP (ChIP) assay

C3H10T1/2 cells were submitted to ChIP assay 48 h after AdGFP or AdBMP9 infection. 80%–90% confluent cells were placed in 10-cm dishes and crosslinked at room temperature for 10 min with 1% formaldehyde before being quenched with glycine (P2078-2, Beyotime, Shanghai, China). DNA fragments from 250 bp to 500 bp were obtained by sonicating the samples. DNA-protein complexes were enriched using the p-Stat3 (Tyr705) primary antibody. Rabbit IgG was used as a negative control. The promoter fragments of *LGR4* were detected by PCR. The PCR primers used for this assay are listed in Table 1.

### Immunofluorescence analysis

4% paraformaldehyde was added to fix C3H10T1/2 cells for 15 min after three PBS washes. Then, the cells were treated with 1% Triton X-100 (9002-93-1, Invitrogen, Carlsbad, California, USA) and blocked with 5% bovine serum albumin (BSA) (ST023, Beyotime, Shanghai, China) in PBS at room temperature for 30 min. Samples were incubated with the primary antibody at 4 °C overnight, followed by 1 h (protected from light) of processing with the secondary antibody coupled to DyLight 488 or 649 (1:100) at 37 °C. After 4′, 6-diamidino-2-phenylindole staining for 5 min, an anti-fluorescence quenching reagent (P0126, Beyotime, Shanghai, China) was used to seal the slices. Images were visualized by confocal microscopy (TCS-SP8 SR, Leica, Wetzlar, Germany).

### Luciferase reporter assay

In a 96-well plate, a complete DMEM medium was used to maintain HEK293 cells (2 × 10^4^ cells/mL). After 24 h, the medium was discarded. Plasmids and transfection reagents were mixed in a blank DMEM medium at room temperature for 10 min. The mixers were added to the plate for 6 h, and the medium was replaced with a complete DMEM medium. All wells were supplemented with pGL3-Basic and pRL-TK vector plasmids. After 48 h, the luciferase activities were measured using the Dual-Glo® Luciferase Assay Kit (E2940, Promega, Beijing, China).

### Calvarial bone defect repair in mice

C3H10T1/2 cells (5 × 10^4^ cells/mL) were seeded in 24-well plates. After 7 days, the medium was pulled out, and each well received an addition of pre-cooled sterile PBS to drive the cells detached with membrane-like attachments. Then, cells were collected and implanted into the bone defect sites within 4 h.

Six groups of mice were randomly assigned (*n* = 6, model, AdGFP, AdBMP9, AdBMP9 plus AdsiLGR4, AdBMP9 plus AdsiRaptor, AdBMP9 and AdsiRaptor plus AdLGR4). Mice were anesthetized with 1% pentobarbital (50 mg/kg) and then intraperitoneally injected with penicillin (1 mg/kg). The hair on the top of the skull was shaved, and the surgical site was disinfected with iodophor. A mouse head was fixed, and a midline sagittal incision was made on the scalp to expose the parietal bone. A circular defect was made approximately 2 mm from the midline on the left side using a 3-mm annular electric drill. To minimize the damage to the skull and the adjacent blood vessels, pre-cooled sterile saline solution was sprayed onto the skull reducing the temperature while drilling. Then, the pre-treated cell membranes were used to fill the defects, and the incision was carefully sutured. Penicillin was injected intraperitoneally for 3 days after the operation. After 8 weeks, mice were anesthetized with 2% pentobarbital sodium intraperitoneally and euthanized with carbon dioxide for further assays.

### Micro-computed tomography (micro-CT) assay

Cranial specimens were scanned using a Bruker Micro-CT Skyscan 1276 system (Kontich, Belgium). Parameters were set as follows: voxel size 6.5 μm, medium resolution, 85 kV, 200 μA, and integration time 384 ms. Density measurements were calibrated to the manufacturer's calcium hydroxyapatite phantom. Analysis was performed using the manufacturer's software. Reconstruction was performed using NRecon software (version 1.7.4.2). Methods based on distance transformation of the grayscale original images (CTvox software, version 3.3.0) were used to obtain 3D images from contoured 2D images.

### Histological evaluation

A 48-h formalin fixation, 15% ethylene diamine tetraacetic acid decalcification, and paraffin embedding process were performed to prepare bone masses. After deparaffinization and rehydration, segments were stained with hematoxylin and eosin (H&E). For immunohistochemical staining, sections were deparaffinized, rehydrated, and blocked with 3% H_2_O_2_. Sections were cleaned using double distilled water, blocked for 20 min with 5% BSA, and incubated with LGR4 primary antibody (1:1000) at 4 °C overnight. Following that, the secondary antibody was applied at room temperature for 1 h before being developed with diaminobenzidine. Images were taken under a microscope (DM4B, Leica).

### Statistical analysis

The data were expressed as mean ± standard deviation (SD). Each assay was repeated at least three times independently. Analysis was performed using SPSS 17.0. One-way analysis of variance or two-tailed student's *t*-tests were used for statistical analysis. A *P*-value less than 0.05 was considered statistically significant.

## Results

### LGR4 expression is down-regulated in the bone tissue of *BMP9*-KO mice

**Figure 1:**
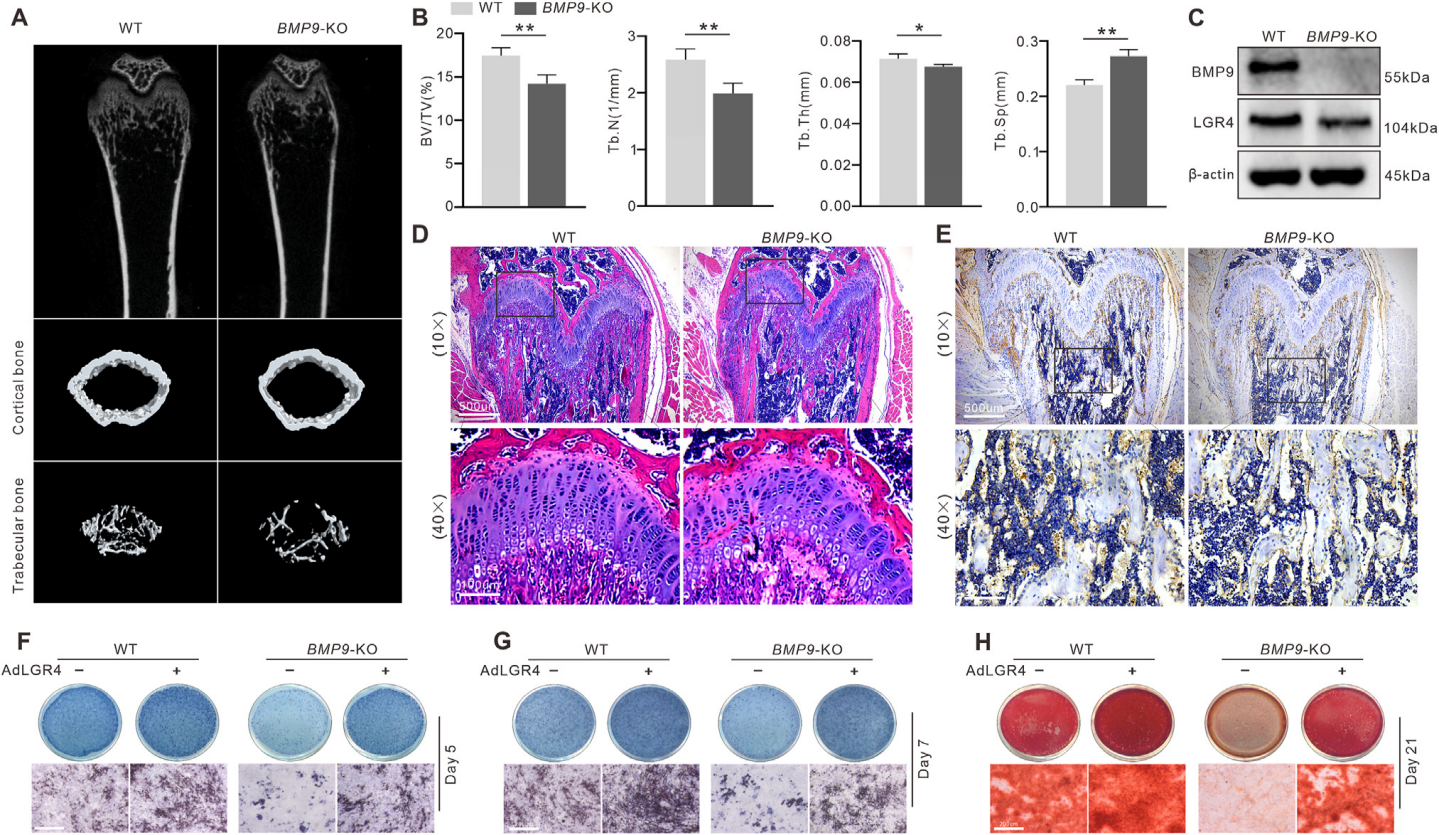
Effect of LGR4 on bone formation in *BMP9*-KO mice. **(A)** The micro-CT images showing BMP9's effect on bone formation. **(B)** Quantitative results of micro-CT assay showing BMP9's effect on bone formation. **(C)** The effect of BMP9 on LGR4 in BMSCs from 4-week-old WT and *BMP9*-KO mice cultured in an osteogenic medium for 7 days shown by Western blotting. **(D)** The effect of BMP9 on bone formation shown by H&E staining. **(E)** The effect of BMP9 on LGR4 in femurs from 4-week-old WT and *BMP9*-KO mice shown by immunohistochemistry staining. **(F, G)** The effect of LGR4 on the ALP activity of BMSCs shown by histochemical staining. **(H)** The effect of LGR4 on the mineralization of BMSCs shown by histochemical staining. BV/TV, the ratio of bone volume to total volume; Tb.N, trabecular number; Tb.Th, trabecular thickness; Tb.Sp, trabecular separation. ^∗^*P* < 0.05, ^∗∗^*P* < 0.01; *n* = 6.

Femoral tissues of 4-week-old WT or *BMP9*-KO mice were subjected to micro-CT analysis as shown in Figure 1A. The *BMP9*-KO mice displayed osteoporosis characterized by reduced bone volume to tissue volume ratio (BV/TV), decreased trabecular number (Tb.N) and trabecular thickness (Tb.Th), and increased trabecular separation (Tb.Sp) when compared with WT mice (Fig. 1B). H&E staining (Fig. 1D) showed that *BMP9*-KO mice exhibited a thinner growth plate than that of WT mice. In addition, the growth plates of *BMP9*-KO mice showed disorganization in the proliferation zone and a delay in the maturation of hypertrophic chondrocytes. Immunohistochemical staining (Fig. 1E) of the femur specimens and Western blot analysis of primary BMSCs (Fig. 1C) both confirmed the reduced LGR4 expression in *BMP9*-KO mice. Primary BMSCs were isolated from mouse bone marrow and subsequently cultured in osteogenic induction media. The ALP staining assay revealed that exogenous LGR4 partially restored the decrease in ALP activity observed on days 5 and 7 following BMP9 deletion (Fig. 1F, G). Moreover, exogenous LGR4 was found to almost restore the decreased mineralization in *BMP9*-KO primary BMSCs after 21 days of incubation in the osteogenic induction medium (Fig. 1H). These observations showed a possible connection between LGR4 and the osteogenic potential of BMP9.

### The effect of BMP9 on LGR4 expression in C3H10T1/2 cells

**Figure 2:**
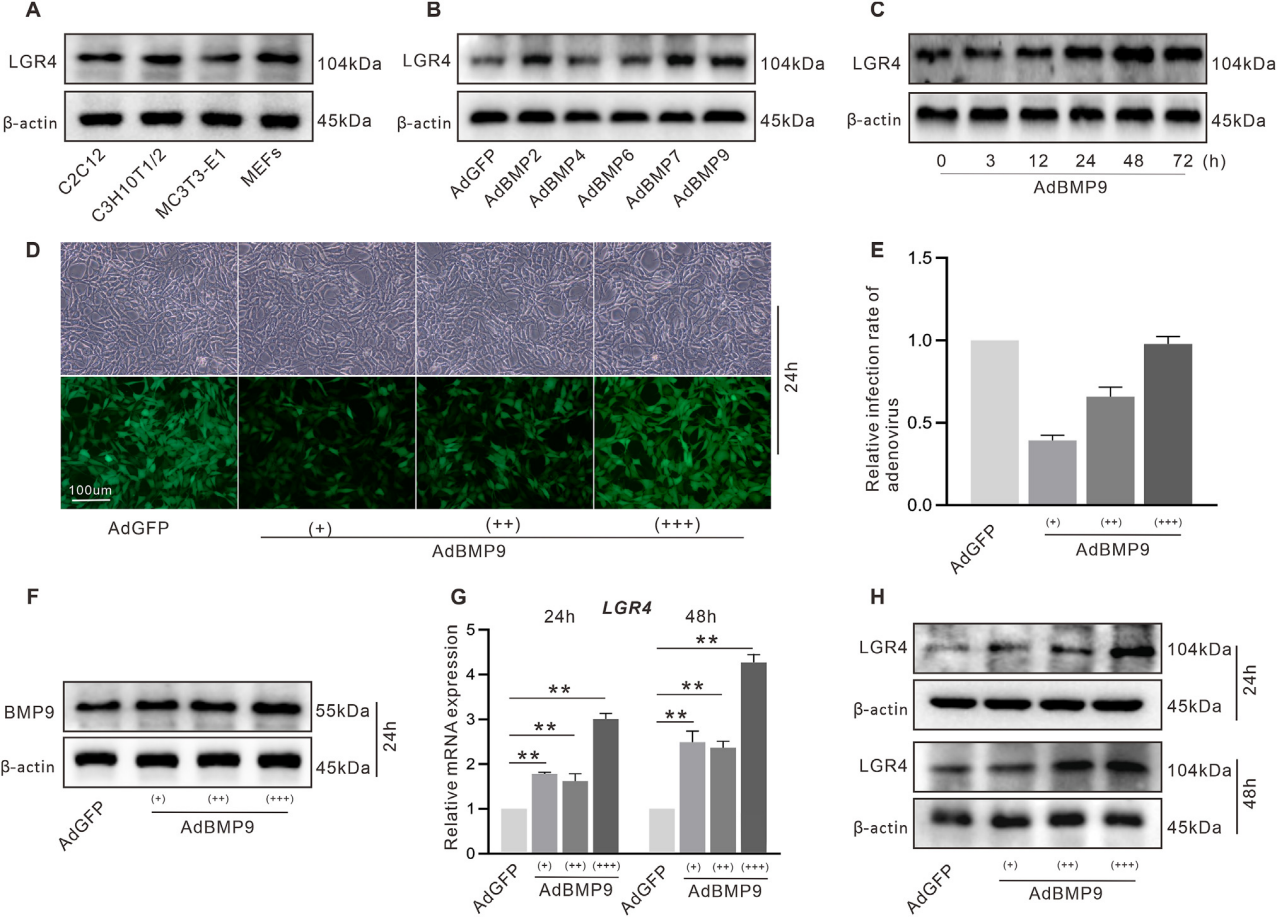
Effect of BMP9 on LGR4 in multiple progenitor cells. **(A)** The level of LGR4 in the progenitor cells shown by Western blotting. **(B)** The effect of osteogenic BMP9 on LGR4 shown by Western blotting. **(C)** The effect of BMP9 on LGR4 over time shown by Western blotting. **(D)** The images of C3H10T1/2 cells showing the transfection rates of BMP9 at 24 h (scale bar, 100 μm; original magnification, ×100). **(E)** Relative quantification of BMP9 adenovirus infection rate in C3H10T1/2 cells. **(F)** Western blotting shows BMP9 recombinant adenovirus affects the protein level of BMP9 at 24 h. **(G)** RT-qPCR assay showing BMP9 affects LGR4 expression. **(H)** Western blotting shows BMP9 affects the protein level of LGR4 at 24 h and 48 h. ^∗^*P* < 0.05, ^∗∗^*P* < 0.01; *n* = 3.

LGR4 is expressed in progenitor cells (Fig. 2A). C3H10T1/2 cell line, which is generated from mouse embryos, is a popular choice for inducing osteogenic differentiation and is considered a valuable model for MSC research in this context. LGR4 protein was elevated in C3H10T1/2 cells by BMP9, BMP2, and BMP7 (Fig. 2B). BMP9 (1 × 10^8^ PFU/mL) increased the LGR4 protein level in a time-dependent manner (Fig. 2C). The infection rate of adenovirus (Fig. 2D, E) and the protein expression of BMP9 (Fig. 2F) in C3H10T1/2 cells confirmed the effectiveness of adenovirus with different titers ((+): 2.5 × 10^7^ PFU/mL; (++): 5 × 10^7^ PFU/mL; (+++): 1 × 10^8^ PFU/mL). The LGR4 mRNA and protein were both increased by BMP9 (Fig. 2G, H). According to these results, LGR4 might contribute to regulating BMP9's osteogenic potency in MSCs.

### The effect of LGR4 on the BMP9-induced osteoblastic markers in C3H10T1/2 cells

**Figure 3:**
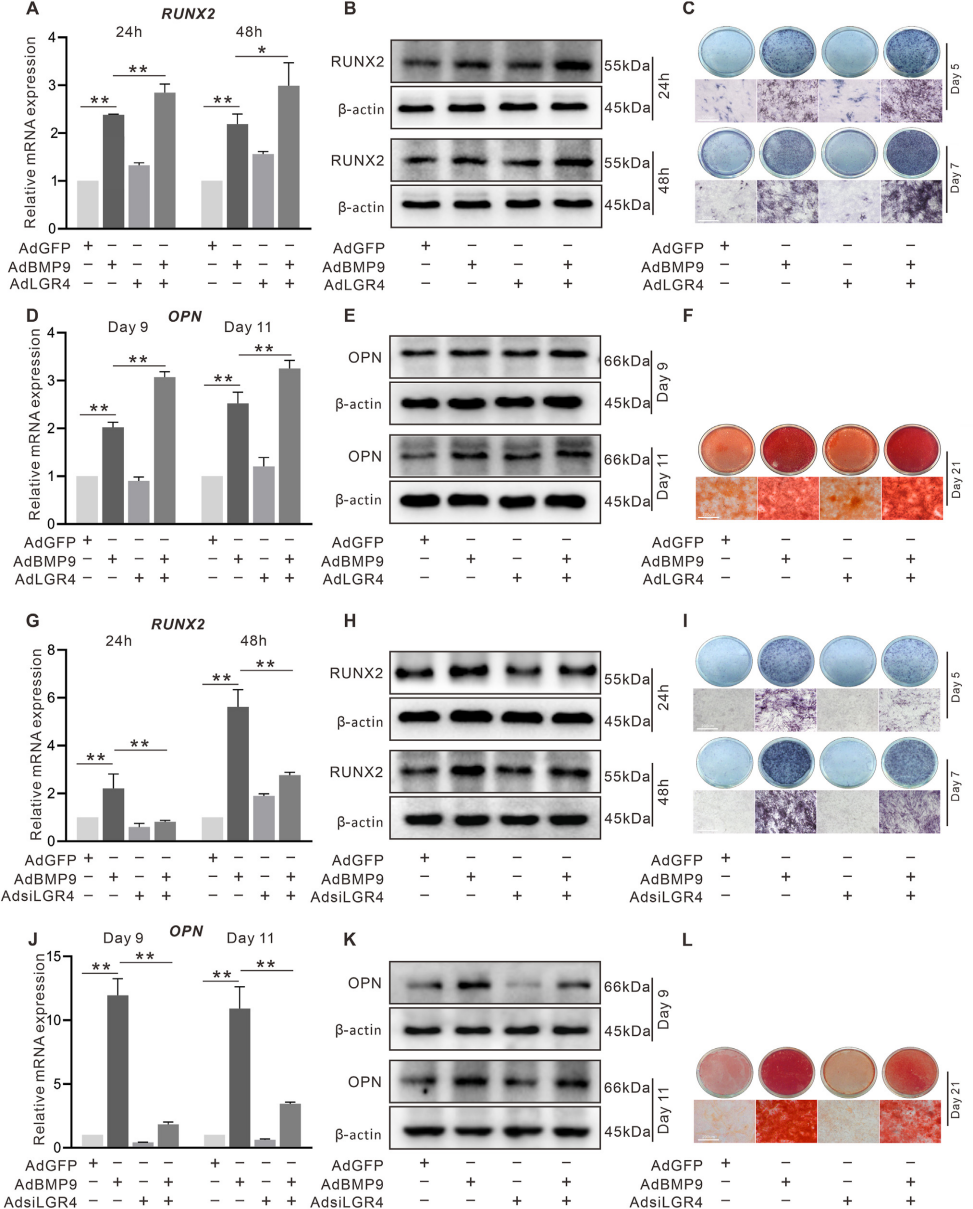
Effect of LGR4 on BMP9-induced osteogenic markers in C3H10T1/2 cells. **(A)** The effects of BMP9 and/or LGR4 on RUNX2 mRNA expression shown by RT-qPCR. **(B)** The effects of BMP9 and/or LGR4 on RUNX2 protein level shown by Western blotting. **(C)** The effects of BMP9 and/or LGR4 on ALP activity shown by alkaline phosphatase assay. **(D)** The effects of BMP9 and/or LGR4 on OPN mRNA expression shown by RT-qPCR. **(E)** The effects of BMP9 and/or LGR4 on OPN level shown by Western blotting. **(F)** The effects of BMP9 and/or LGR4 on mineralization shown by alizarin red S staining. **(G)** The effects of BMP9 and/or LGR4 knockdown on RUNX2 mRNA expression shown by RT-qPCR. **(H)** The effects of BMP9 and/or LGR4 knockdown on RUNX2 protein level shown by Western blotting. **(I)** The effects of BMP9 and/or LGR4 on ALP activity shown by alkaline phosphatase assay. **(J)** The effects of BMP9 and/or LGR4 knockdown on OPN mRNA expression shown by RT-qPCR. **(K)** The effects of BMP9 and/or LGR4 knockdown on OPN protein level shown by Western blotting. **(L)** The effects of BMP9 and/or LGR4 on mineralization shown by alizarin red S staining. ^∗^*P* < 0.05, ^∗∗^*P* < 0.01; *n* = 3.

The BMP9 effect on the mRNA and protein level of RUNX2 (Fig. 3A, B) and OPN (Fig. 3D, E) were significantly increased by exogenous LGR4 and reduced upon LGR4 knockdown (Fig. 3G, H, J, and K). Furthermore, the BMP9-induced ALP activity and mineralization were both enhanced by LGR4 (Fig. 3C, F) and inhibited by LGR4 knockdown (Fig. 3I, L). These data indicated that LGR4 may be associated with the BMP9 osteogenic capability.

### The effect of mTOR on BMP9-boosted osteoblastic markers in C3H10T1/2 cells

**Figure 4:**
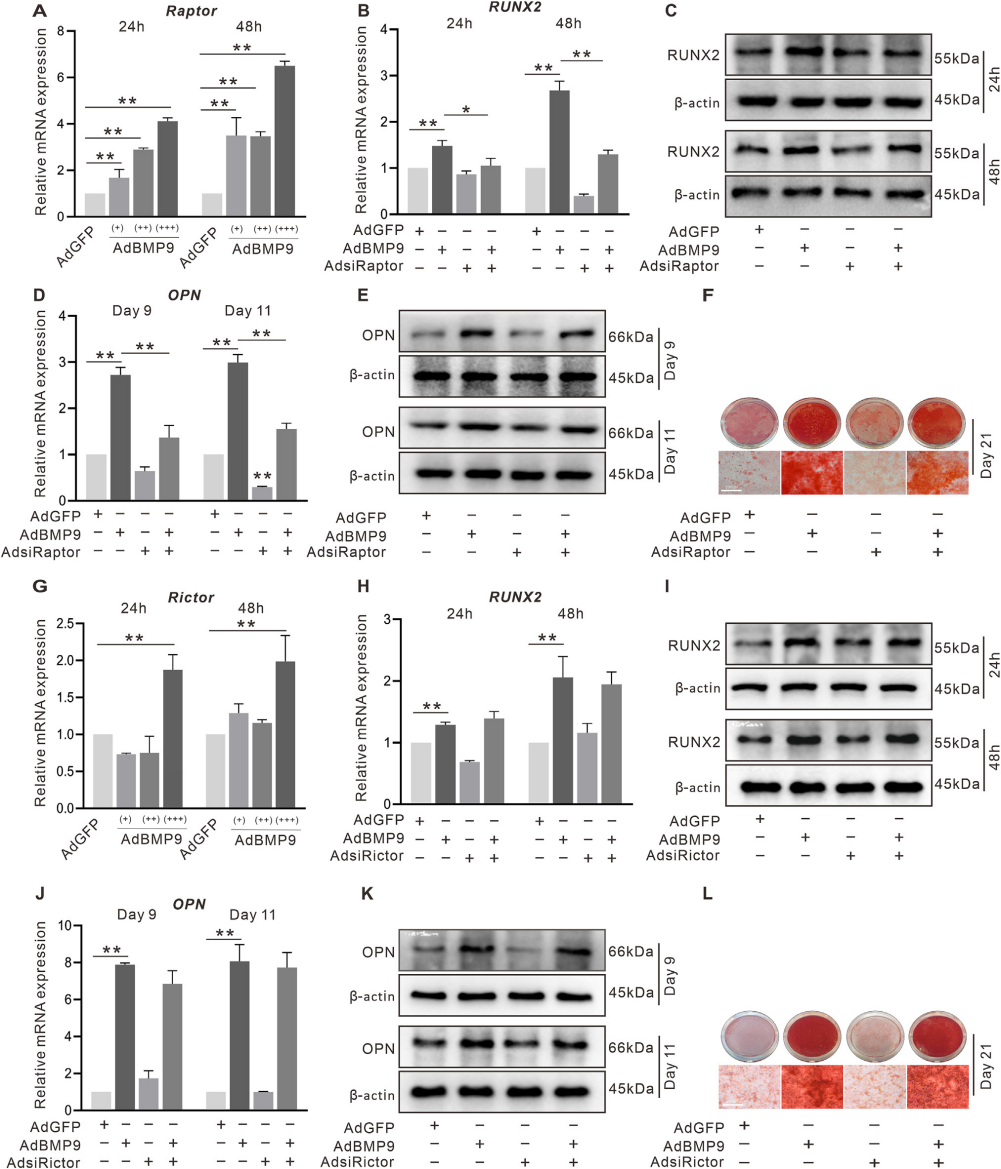
Effect of mTOR on BMP9-induced osteogenic markers in C3H10T1/2 cells. **(A)** The effect of BMP9 on Raptor shown by RT-qPCR. **(B)** The effects of BMP9 and/or Raptor knockdown on RUNX2 mRNA expression shown by RT-qPCR. **(C)** The effects of BMP9 and/or Raptor knockdown on RUNX2 protein level shown by Western blotting. **(D)** The effects of BMP9 and/or Raptor knockdown on OPN mRNA level shown by RT-qPCR. **(E)** The effects of BMP9 and/or Raptor knockdown on OPN protein level shown by Western blotting. **(F)** The effects of BMP9 and/or Raptor knockdown on mineralization shown by alizarin red S staining. **(G)** The effect of BMP9 on Rictor mRNA level shown by RT-qPCR. **(H)** The effects of BMP9 and/or Rictor on RUNX2 mRNA level shown by RT-qPCR. **(I)** The effects of BMP9 and/or Rictor knockdown on RUNX2 protein level shown by Western blotting. **(J)** The effects of BMP9 and/or Rictor knockdown on OPN mRNA level shown by RT-qPCR. **(K)** The effects of BMP9 and/or Rictor knockdown on OPN protein level shown by Western blotting. **(L)** The effects of BMP9 and/or Rictor knockdown on mineralization shown by alizarin red S staining. ^∗^*P* < 0.05, ^∗∗^*P* < 0.01; *n* = 3.

PI3K/Akt/mTOR pathway plays an important role in regulating the osteoblastic differentiation spurred by BMP9.^30^ However, the precise regulatory roles of mTORC1 and mTORC2 in this process remain unclear. PCR results revealed that Raptor and Rictor were both up-regulated by BMP9 (Fig. 4A, G). The BMP9-induced RUNX2 and OPN were decreased by Raptor knockdown (Fig. 4B–E); by contrast, it was not changed by Rictor knockdown (Fig. 4H–K). Similar results were observed in matrix mineralization; Raptor knockdown markedly inhibited BMP9-induced mineralization (Fig. 4F), whereas Rictor knockdown had no apparent effect (Fig. 4L). These findings indicated that it was mTORC1 rather than mTORC2 which mediated the BMP9-induced osteogenic differentiation.

### The effects of LGR4 and/or Raptor knockdown on osteoblastic and adipogenic markers induced by BMP9 in C3H10T1/2 cells

**Figure 5:**
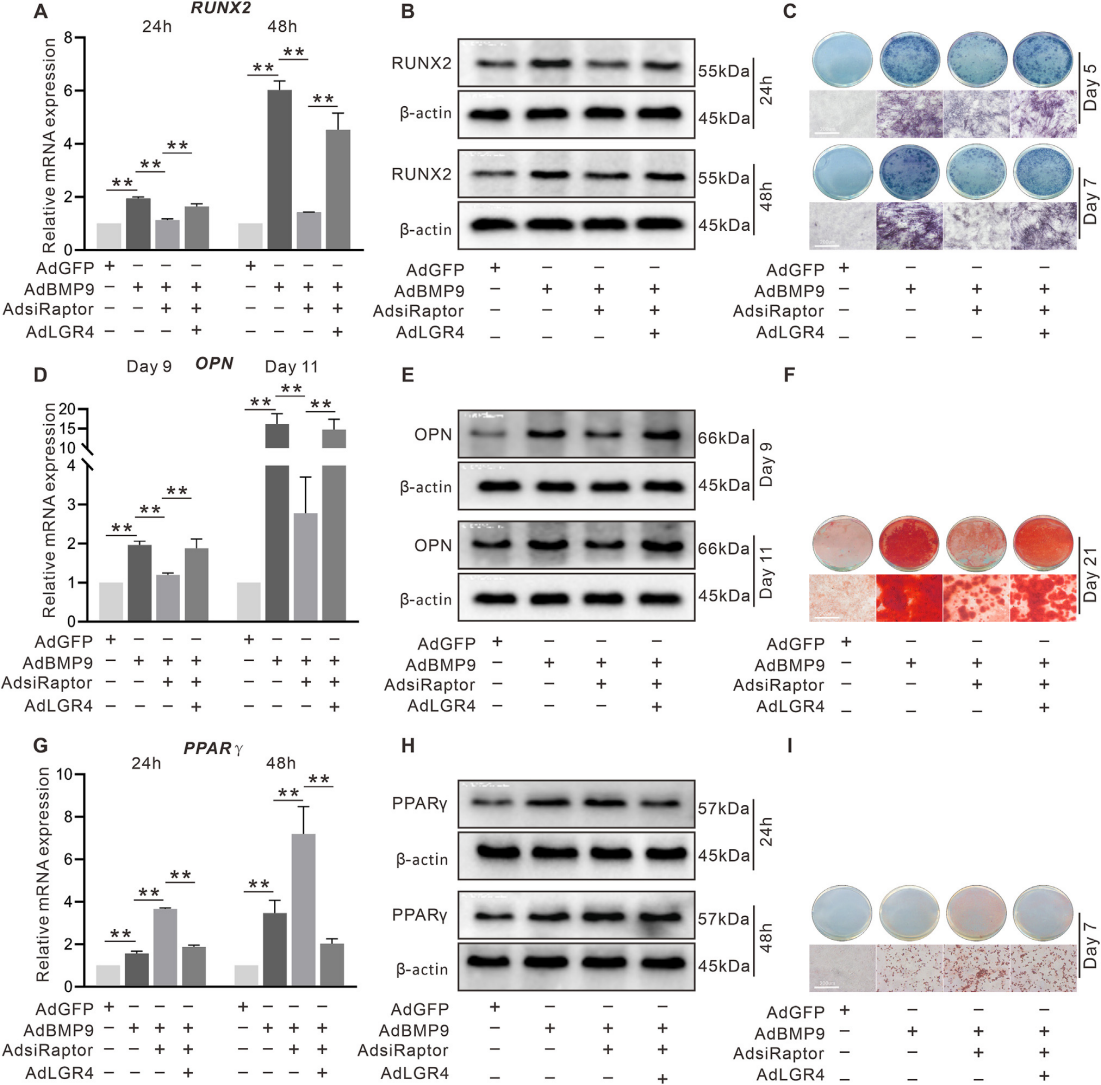
Effect of LGR4 and/or Raptor knockdown on osteoblastic and adipogenic markers induced by BMP9 in C3H10T1/2 cells. **(A)** The effects of LGR4 and/or Raptor knockdown on BMP9-induced RUNX2 mRNA level shown by RT-qPCR. **(B)** The effects of LGR4 and/or Raptor knockdown on BMP9-induced RUNX2 shown by Western blotting. **(C)** The effects of LGR4 and/or Raptor knockdown on BMP9-induced ALP activity shown by histochemical staining. **(D)** The effects of LGR4 and/or Raptor knockdown on BMP9-induced OPN mRNA level shown by RT-qPCR. **(E)** The effects of LGR4 and/or Raptor knockdown on BMP9-induced OPN level shown by Western blotting. **(F)** The effects of LGR4 and/or Raptor knockdown on BMP9-induced matrix mineralization shown by histochemical staining. **(G)** The effects of LGR4 and/or Raptor knockdown on BMP9-induced PPARγ mRNA expression shown by RT-qPCR. **(H)** The effects of LGR4 and/or Raptor knockdown on BMP9-induced PPARγ protein level shown by Western blotting. **(I)** The effects of LGR4 and/or Raptor knockdown on BMP9-induced lipid droplets formation shown by oil red O staining. ^∗^*P* < 0.05, ^∗∗^*P* < 0.01; *n* = 3.

Raptor knockdown suppressed the BMP9 effect on increasing RUNX2 (Fig. 5A, B) and OPN (Fig. 5D, E), which was reversed by LGR4. Similarly, the BMP9-induced ALP activity and mineralization were reduced by Raptor knockdown (Fig. 5C, F), which were obviously counteracted by LGR4. It is well known that osteoblastic differentiation may be promoted at the expenditure of adipogenesis.^31^, ^32^, ^33^ LGR4 is involved in regulating adipogenesis homeostasis.^34^^,^^35^ Disruption of the mTORC1 signal induced an increase in adipose tissue and a decrease in bone mass in mice.^36^ Given that BMP9 is capable of inducing osteogenesis and adipogenesis simultaneously,^8^^,^^37^ we determined the LGR4 and/or Raptor effect on the BMP9-induced lipogenic differentiation in MSCs. According to PCR and Western blot analyses, the BMP9-induced PPARγ was amplified by silencing Raptor, which was nearly abrogated by LGR4 (Fig. 5G, H). Similarly, BMP9-induced lipid droplet accumulation was enhanced by silencing Raptor, which was reversed by LGR4 (Fig. 5I). These results suggested that mTORC1 may promote the BMP9 osteoblastic potential through reducing adipogenesis via LGR4.

### The effect of LGR4 and Raptor on skull defect repair promoted by BMP9

**Figure 6:**
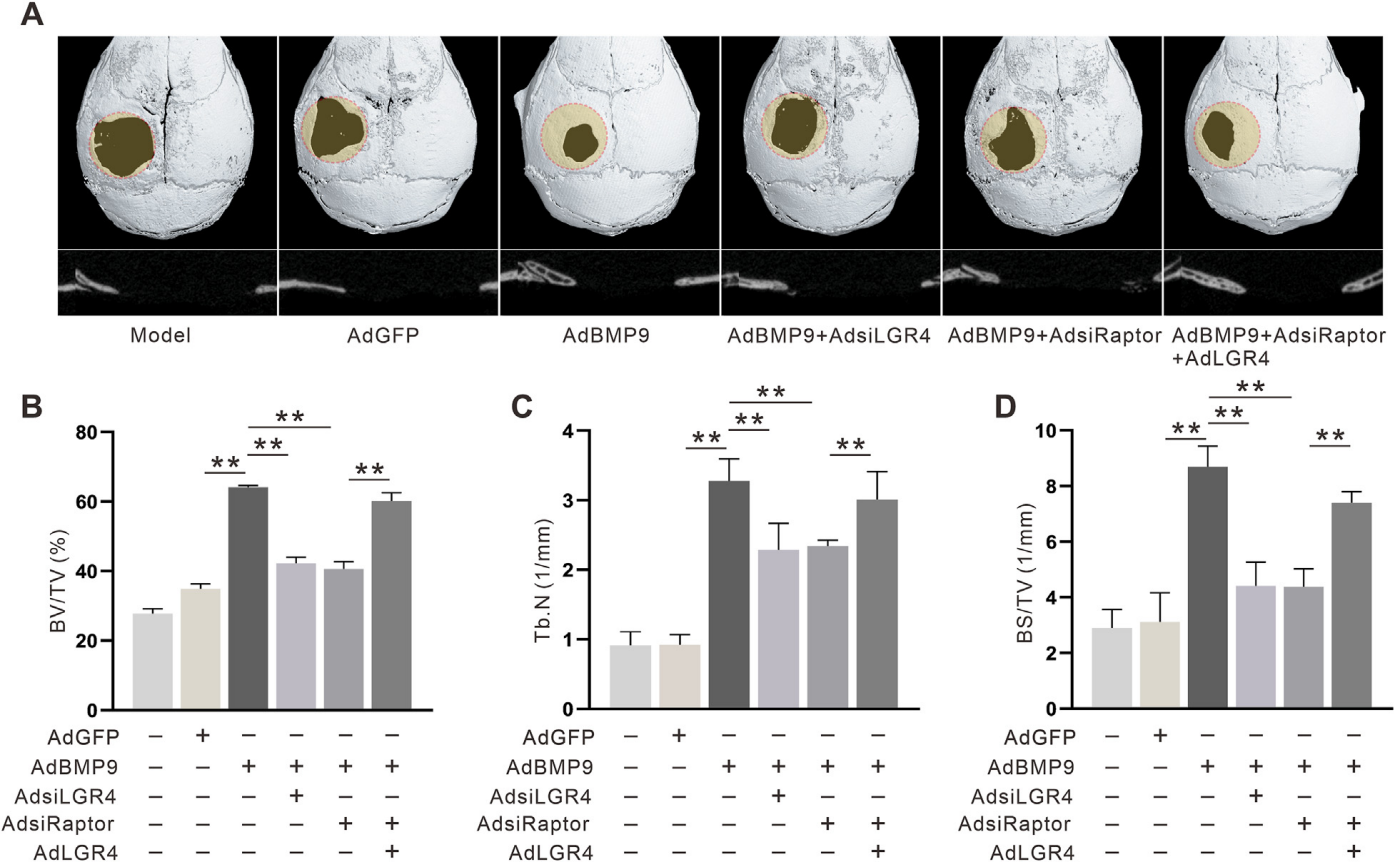
Effect of LGR4 and Raptor on the BMP9-induced skull defect repair. **(A)** The effect of LGR4 and Raptor knockdown on the bone formation induced by BMP9 (the area circled in red is the area of the defect site) shown by reconstruction of micro-CT analysis. **(B–D)** The effects of LGR4 and Raptor knockdown on bone formation induced by BMP9 indicated by quantitative results of the micro-CT assay of BV/TV, Tb.N, and BS/TV. BV/TV, bone volume per tissue volume; Tb.N, trabecular number; BS/TV, bone volume per bone surface area. ^∗^*P* < 0.05, ^∗∗^*P* < 0.01 *vs.* AdGFP control group; ^#^*P* < 0.05, ^##^*P* < 0.01 *vs.* AdBMP9 group; ^Δ^*P* < 0.05, ^ΔΔ^*P* < 0.01 *vs.* AdBMP9 + AdsiRaptor group; *n* = 6.

Micro-CT images (Fig. 6A) and analysis of skeletal parameters (Fig. 6B–D) including bone volume per tissue volume (BV/TV), trabecular number (Tb.N), and bone volume per bone surface area (BS/TV) showed that by week 8, the control group exhibited limited new bone formation around the defect site, while BMP9 significantly increased new bone formation in this region. However, the knockdown of either LGR4 or Raptor attenuated the reparative effects of BMP9. The inhibitory effect of Raptor knockdown on the BMP9-induced defect repair was partially reversed by LGR4. These results indicated that Raptor may promote the osteoblastic capability of BMP9 via LGR4.

### The effect of mTORC1/Stat3 signal on BMP9-induced LGR4 expression and osteogenesis in C3H10T1/2 cells

**Figure 7:**
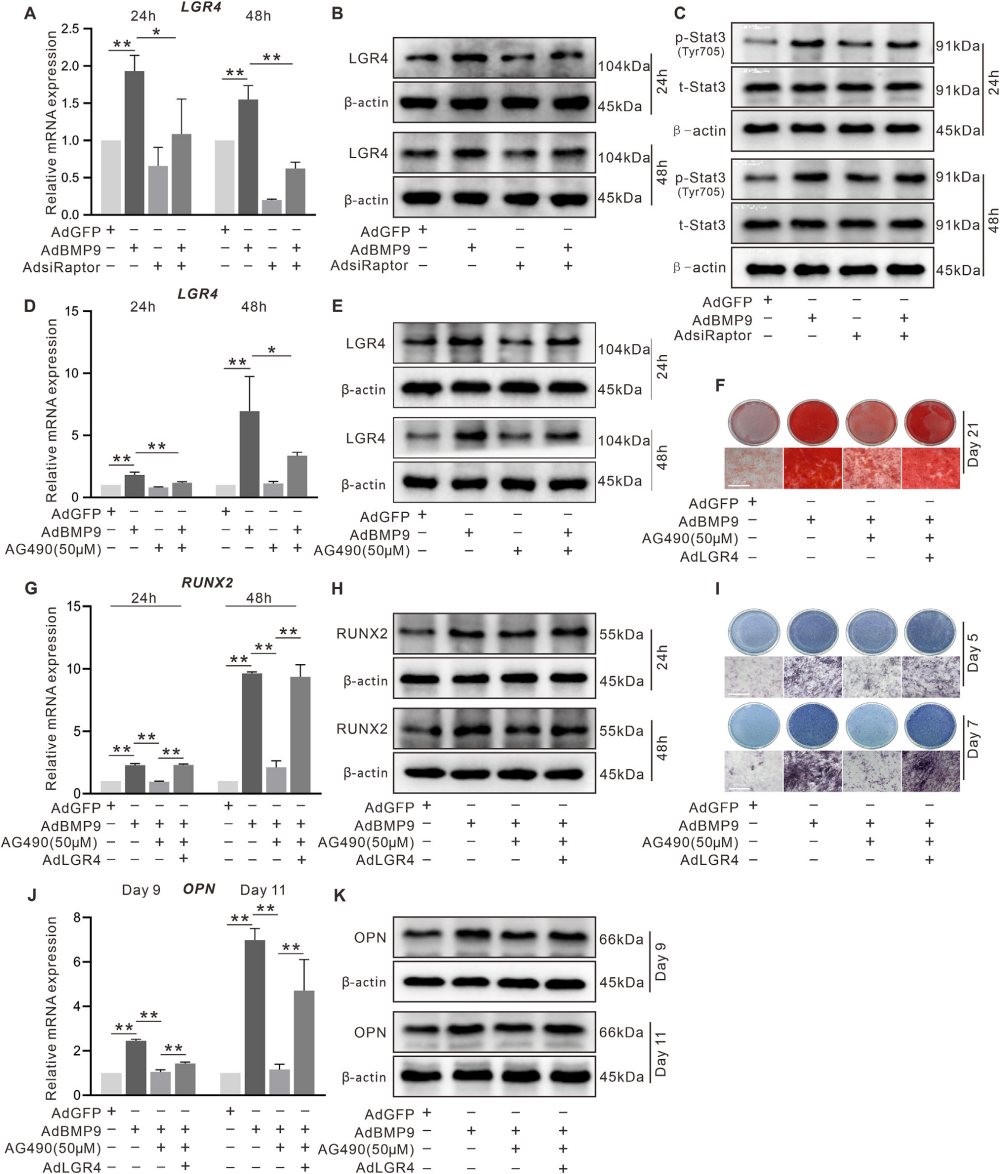
Effect of mTORC1/Stat3 signal and LGR4 on BMP9-induced osteogenesis in C3H10T1/2 cells. **(A)** The effects of BMP9 and/or Raptor knockdown on LGR4 mRNA expression shown by RT-qPCR. **(B)** The effects of BMP9 and/or Raptor knockdown on LGR4 protein level shown by Western blotting. **(C)** The effects of BMP9 and/or Raptor knockdown on total and phosphorylated Stat3 levels shown by Western blotting. **(D)** The effects of AG490 and/or BMP9 on LGR4 mRNA shown by RT-qPCR. **(E)** The effects of AG490 and/or BMP9 on LGR4 shown by Western blotting. **(F)** The effects of AG490 and/or LGR4 on BMP9-induced mineralization shown by histochemical staining. **(G)** The effects of AG490 and/or LGR4 on BMP9-induced RUNX2 mRNA expression shown by RT-qPCR. **(H)** The effects of AG490 and/or LGR4 on BMP9-induced RUNX2 shown by Western blotting. **(I)** The effects of AG490 and/or LGR4 on BMP9-induced ALP activity shown by histochemical staining. **(J)** The effects of AG490 and/or LGR4 on BMP9-induced OPN mRNA expression shown by RT-qPCR. **(K)** The effects of AG490 and/or LGR4 on BMP9-induced OPN shown by Western blotting. AG490: Jak2/Stat3 inhibitor. ^∗^*P* < 0.05, ^∗∗^*P* < 0.01; *n* = 3.

Analysis by RT-qPCR and Western blotting (Fig. 7A, B) showed that knockdown of Raptor reduced LGR4 expression induced by BMP9. Notably, the biological effects of BMPs have been previously reported to be mediated by the phosphorylation of Stat3 (p-Stat3).^38^^,^^39^ In addition, Stat3 signaling could be regulated by PI3K/Akt/mTOR.^40^^,^^41^ Based on this, we next determined whether Raptor and BMP9 could modulate the phosphorylation of Stat3. The results showed that BMP9 raised p-Stat3 level, which was suppressed by silencing Raptor; BMP9 and Raptor exhibited no apparent effect on total protein levels of Stat3 (Fig. 7C). Further analysis showed that the BMP9-induced LGR4 was reduced by AG490, a classical JAK/Stat3 inhibitor (Fig. 7D, E). Previous studies have demonstrated that AG490 can impair osteogenic differentiation of MSCs and reduce bone formation in mice.^42^ Given that LGR4 expression was reduced by AG490, we next investigated whether AG490 and LGR4 might affect BMP9-boosted osteoblastic markers in C3H10T1/2 cells. It was found that the BMP9-induced RUNX2 (Fig. 7G, H) or OPN (Fig. 7J, K) were reduced by AG490, which was almost completely reversed by LGR4. Similarly, the BMP9-induced ALP activity (Fig. 7I) and mineralization (Fig. 7F) were reduced by AG490, which was also reversed by LGR4. These findings indicated that BMP9 may regulate LGR4 expression via the Raptor/Stat3 signaling.

### The effect of mTORC1/Stat3 signal on regulating LGR4 expression

**Figure 8:**
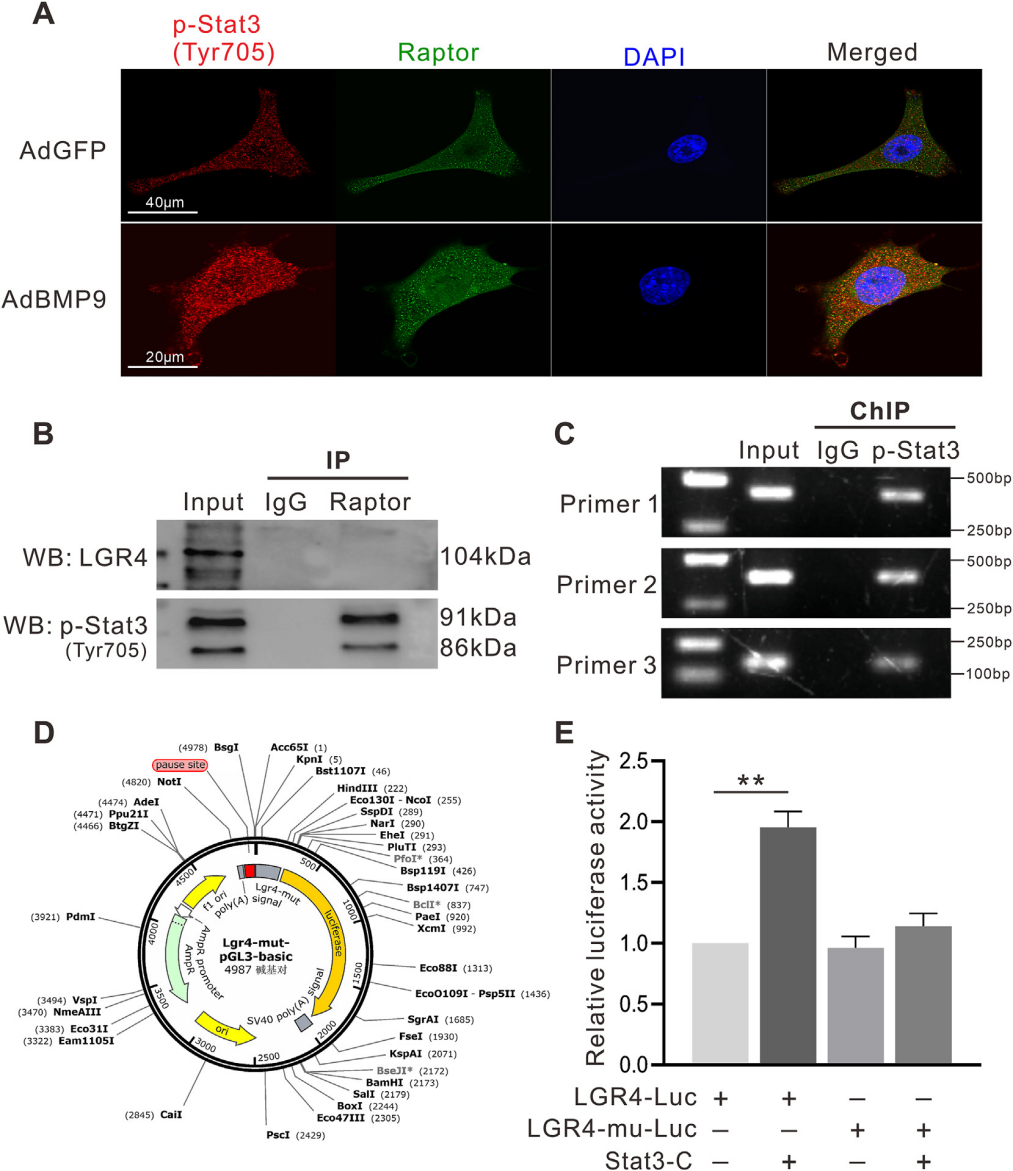
Regulation mechanism of mTORC1/Stat3 signal on *LGR4* expression. **(A)** The location of Raptor or p-Stat3 in C3H10T1/2 cells shown by confocal assay. **(B)** IP assay shows Raptor interacts with p-Stat3 in C3H10T1/2 cells. **(C)** The possible interaction between p-Stat3 and *LGR4* promoter shown by ChIP assay. **(D)** The schematic diagram showing the construction of LGR4 luciferase reporter. **(E)** The effect of p-Stat3 on *LGR4* promoter activity shown by luciferase reporter assay (^∗∗^*P* < 0.01; *n* = 6).

Immunofluorescence and IP analysis confirmed that Raptor interacts with Stat3, and BMP9 facilitates this interaction (Fig. 8A, B). As a transcription factor, Stat3 can regulate the expression of multiple target genes. We analyzed the promoter region of *LGR4* using *JASPAR* (*genereg.net*) and found that a potential binding site for Stat3 exists, which suggested that Stat3 may regulate LGR4 expression directly. The ChIP assay showed that LGR4 promoter fragments could be enriched by anti-LGR4 antibodies (Fig. 8C). The LGR4 luciferase reporter (LGR4-luc) and the mutant *LGR4* reporter with the predicted Stat3 binding site deleted (LGR4-mu-luc) were constructed to verify the regulatory effect of p-Stat3 on LGR4 promoter activity (Fig. 8D). In addition, HEK293 cells were transfected with LGR4-mu-luc and the Stat3 active vector (Stat3-C). As shown in Figure 8E, Stat3 increased *LGR4* promoter activity, whereas mutation of the Stat3-binding site in the *LGR4* promoter abolished this effect. These data demonstrated that Stat3 can regulate the promoter activity of *LGR4*.

## Discussion

BMP9 has emerged as a promising candidate for regenerative medicine, however, its osteogenic potential needs to be optimized. The Wnt/β-catenin signal is known to play an important role in BMP9 osteoblastic potential, but details underlying BMP9-promoted activation of this pathway remain unclear. In the present study, we demonstrated that BMP9 up-regulated LGR4, and the BMP9 osteogenic potency was promoted by LGR4 but reduced by LGR4 knockdown. Regarding the mechanism, we found that the BMP9 osteogenic potential was reduced by silencing Raptor; BMP9 enhanced Stat3 phosphorylation and promoted the interaction between Raptor and p-Stat3, and *LGR4* promoter activity was enhanced by Stat3. Our findings indicated that BMP9 may promote osteoblastic development by activating the Wnt/β-catenin signal through up-regulating LGR4 expression via the mTORC1/Stat3 signal, at least.

Bone regeneration with MSCs as seed cells provides a basis for the clinical treatment of bone defects. MSCs can initiate lineage commitment under the regulation of specific factors. For example, the transcription factor RUNX2 is known to drive osteogenic lineages,^43^ while PPARγ promotes adipogenic lineage.^44^ To promote bone defect healing effectively, efforts to enhance osteoblastic lineage commitment must be adopted, and BMP9 is one of the promising candidates for achieving this goal. Wnt/β-catenin is essential for promoting bone growth, and inhibition of this pathway could enhance adipogenesis while reducing osteogenesis.^45^^,^^46^ It is well known that the Wnt/β-catenin signal could be activated by BMP9, and β-catenin knockdown significantly reduced the BMP9 osteoblastic potential.^12^ Therefore, increasing Wnt/β-catenin signaling activity may contribute to augmenting the BMP9 osteoblastic potential.

The canonical Wnt pathway can be activated by receptors (frizzled family) and co-receptors (lipoprotein receptor-related protein family).^47^ The LGRs, when binding to R-spondins (RSPOs), form a complex that sequesters ZNF/RNF43 and increase the amount of membrane-available frizzled proteins.^48^ LGR4 is widely expressed in various tissues and is expressed abundantly in bone.^49^ Several studies have highlighted the significant role of LGR4, a crucial Wnt/β-catenin regulator, in regulating BMSC differentiation as well as bone development and homeostasis.^19^^,^^50^ Abnormal LGR4 expression was observed in a variety of bone loss diseases.^16^ *LGR4*-KO mice have been found to exhibit delayed osteogenic differentiation and mineralization.^17^ Moreover, BMP-2 up-regulated LGR4 expression in osteoblasts, and loss of LGR4 significantly delayed fracture healing *in vivo* upon BMSC transplantation.^51^ To date, the specific molecular mechanism of LGR4 in regulating osteoblastic differentiation remains unclear in MSCs. Our data revealed that the femur growth and LGR4 expression were both inhibited in *BMP9*-KO mice, and the reduced osteogenic potential of primary BMSCs from *BMP9*-KO mice was partially rescued by exogenous LGR4. BMP9's effect on inducing osteoblastic markers and bone formation was increased by LGR4 but reduced by LGR4 knockdown. These data indicated that BMP9 osteoblastic potency might partially be regulated by LGR4.

PI3K/AKT/mTOR signal is associated with various biological processes, including bone formation and reconstruction.^52^^,^^53^ Activation of this pathway promoted the pre-osteoblasts and BMSCs differentiate into osteoblasts, whereas targeted inhibition suppressed the bone formation *in vivo* and *in vitro*.^54^ BMP9 osteogenic potency can be promoted by activating PI3K/AKT signal,^30^^,^^55^ although the detailed molecular mechanism required further analysis. As downstream targets of PI3K/AKT, both mTOR and GSK-3β contribute to cell growth regulation and protein synthesis. BMP9-induced osteoblastic markers could be promoted by GSK-3β inhibition through activating PI3K/AKT in MC3T3-E1 cells.^24^ However, the effect of mTOR on the BMP9 osteoblastic potency remains unclear. mTOR carries out its functions via two distinct complexes, mTORC1 and mTORC2, which have different biological functions. mTORC1 is primarily responsible for regulating protein synthesis and cell growth, while mTORC2 most often participates in regulating cell metabolism by phosphorylating AGC kinases, such as Akt.^27^ To better understand the roles of mTORC1 and mTORC2 in BMP9-boosted osteogenesis, we inhibited the function of mTORC1 or mTORC2 by blocking the core units Raptor or Rictor, respectively. Our findings indicated that inhibition of mTORC1, but not mTORC2, disrupted the BMP9-induced osteoblastic markers. Thus, mTORC1 may be involved in regulating the BMP9 osteogenic potency. Moreover, Raptor silencing dramatically reduced LGR4 expression and exogenous LGR4 partially reversed the inhibitory effects of Raptor knockdown on BMP9-induced osteoblastic markers. This provides evidence that the LGR4 expression induced by BMP9 may be mediated by mTORC1.

PPARγ is recognized as the key inducer of adipogenesis, while Wnt/β-catenin is defined as the major regulator pathway of osteogenesis.^46^ Therefore, unraveling the interplay between PPARγ and Wnt/β-catenin in MSC fate determination is essential for healthcare, from regenerative medicine to obesity-related issues. Although Raptor and LGR4 are involved in adipogenesis, their potential roles in adipogenic differentiation induced by BMP9 remain unclear. Our findings showed that LGR4 or Raptor knockdown promoted the BMP9 potency of adipogenesis in MSCs, whereas the effect of Raptor knockdown on amplifying adipogenesis was almost eliminated by LGR4. These results suggested that the effect of LGR4 on promoting BMP9 osteoblastic potency may be responsible for reduced adipogenesis, and BMP9 may regulate LGR4 expression via mTORC1/Raptor.

Stat3 is a transcriptional factor with numerous biological functions and is involved in signal regulation in different cell types.^56^ Stat3 is a major regulator of mouse embryonic stem cell fate and a limiting factor for human cell reprogramming.^57^ In addition, Stat3 activation promotes osteogenic differentiation, and Stat3 mutation in humans causes symptoms of autosomal dominant hyper-immunoglobulin E syndrome; bone deformity accompanied by osteoporosis and spontaneous fractures have been observed when Stat3 was conditionally knocked out in osteoblasts.^42^ Stat3 can be activated via the phosphorylation of Tyr705 by an upstream kinase, after which it forms a dimer by the interaction of the two monomers with photoglycan alcohol-Src homology 2, moves to the nucleus, and then interacts with importins to trigger the downstream targets transcription.^58^ Numerous signals can activate Stat3, and the mTOR and Stat3 crosstalk contribute to regulating cell proliferation and differentiation.^40^^,^^41^ We demonstrated that BMP9 increased Stat3 phosphorylation, which was inhibited by silencing Raptor. Raptor can interact with p-Stat3, and the interaction was enhanced by BMP9. Thus, the effect of mTORC1 on LGR4 may be mediated through interacting with p-Stat3. A previous study showed that LGR4 could be up-regulated by Stat3 in human osteosarcoma cells, but reduced when Stat3 was deficient.^59^ Our data demonstrated that Stat3 inhibition with AG490 diminished LGR4 expression, as well as the effect of BMP9 on increasing osteogenic markers. All these results indicated that Stat3 may be a potential mediator of the mTORC1 regulatory effect on LGR4 during the osteoblastic differentiation triggered by BMP9. Moreover, data from ChIP and luciferase reporter assays indicated that Stat3 could bind to the *LGR4* promoter. Thus, the effect of mTORC1 on LGR4 expression may be mediated by Stat3.

In summary, we found that the osteoblastic potency of BMP9 can be enhanced by LGR4, and BMP9 may up-regulate LGR4 through the mTORC1/Stat3 signal. Our findings may provide a new way for BMP9 to activate the Wnt/β-catenin signal in MSCs.

## Ethics declaration

All animal experiments were approved by The Ethics Committee of Chongqing Medical University (No. 2022030).

## Author contributions

Jie Zhang, Yuxi Su, and Baicheng He designed the project. Jie Zhang, Jinhai Jiang, and Hang Liu established the calvarial bone defect repair model in mice. Jie Zhang, Shiyu Wang, Kaixing Ke, Siyuan Liu, Yue Jiang, Lu Liu, and Xiang Gao collected the *in vitro* data. Jie Zhang wrote the manuscript. Yuxi Su and Baicheng He revised the manuscript. All authors read and approved the manuscript.

## Conflict of interests

The authors declare no competing financial interest.

## Funding

This work was supported by 10.13039/501100002865Chongqing Science and Technology Commission project (China) (No. cstc2018jcyjAX0143 to Yuxi Su) and CQMU Program for Youth Innovation in Future Medicine (China) (No. W0154 to Baicheng He).

## Data availability

The datasets generated and analyzed during the current study are available from the corresponding author upon reasonable request.
